# Association of bovine uterine involution disturbances with serum neuropeptide concentrations

**DOI:** 10.14202/vetworld.2020.1854-1857

**Published:** 2020-09-11

**Authors:** Aida Abultdinova, Isatay Jakupov, Joachim Roth, Klaus Failing, Axel Wehrend, Marlene Sickinger

**Affiliations:** 1 Department of Veterinary Medicine, Faculty of Veterinary Medicine and Technology of Animal Husbandry, S. Seifullin Kazakh Agro Technical University, Nur-Sultan, Kazakhstan; 2 Institute of Veterinary Physiology, Justus Liebig University Giessen, Germany; 3 Biomathematics and Data Processing Unit, Justus Liebig University Giessen, Germany; 4 Clinic for Obstetrics, Gynecology and Andrology of Large and Small Animals with Veterinary Ambulance, Justus Liebig University Giessen, Germany; 5 Clinic for Ruminants Internal Medicine and Surgery, Justus Liebig University Giessen, Germany

**Keywords:** dairy cow, diagnostics, inflammation, neuropeptides, uterus involution

## Abstract

**Background and Aim:**

Puerperal diseases influence fertility and should be diagnosed as soon as possible. This study aimed to evaluate the applicability of serum concentrations of substance P (SP), vasoactive intestinal polypeptide (VIP), and interleukin (IL)1β in the early diagnosis of uterine involution disturbances.

**Materials and Methods:**

Blood serum samples of 86 dairy cows from six different farms were harvested within the first 20 days after calving from cows with uterine involution disturbances and healthy controls, respectively. Serum concentrations for SP, VIP, and IL-1β were determined using commercially available ELISA test kits. Statistical analyses included timely changes in blood serum levels and group comparisons of healthy cows and cows with uterine disease.

**Results:**

SP concentrations increased significantly within 20 days after calving (p<0.04) with no significant difference observed between the groups. Moreover, no significant differences were found between VIP and log IL-1β.

**Conclusion:**

Results showed that none of the examined serum parameters seems suitable as indicator of uterine involution disorders. Due to the timely changes in serum concentrations of SP after calving, a correlation to diseases might not be precluded. Further research is needed as regards the establishment of normative values concerning this parameter.

## Introduction

Postpartum uterine diseases result in infertility or subfertility and have high economic significance [1]. A literature-based calculation of the direct costs of treatment reduced milk yield and subfertility associated with uterine disease revealed economic losses of up to £ 16 million/year in the UK [1].

The incidence of metritis (pyrexia up to 10 days postpartum, purulent vaginal discharge, and delayed uterus involution) and clinical endometritis (purulent vaginal discharge for 21 days or more postpartum and delayed uterus involution) is 18.5-40% and 10-20%, respectively [1]. In the past years, several diagnostic possibilities have been developed and validated [2]. Among these traditional diagnostic measures are the transrectal palpation of the uterus, ultrasonography, and vaginal discharge evaluation [2]. At present, studies have focused on pro-inflammatory factors and the uterine and vaginal microbiome associated with puerperal diseases [3-5]. These studies have focused on the local situation within the endometrium, whereas another study determined a possible association between serum pro-inflammatory and anti-inflammatory cytokines and uterine diseases in postpartum dairy cows [6]. In addition to inflammation that is associated with the release of fetal membranes [7], myometrium contractility is essential for a physiological uterine involution [8,9]. Therefore, this study examined serum concentrations of the motility-influencing neuropeptides substance P (SP) and vasoactive intestinal polypeptide (VIP) and the cytokine concentrations of interleukin (IL)-1β.

Single blood tests that might enable the local veterinarian to determine the puerperal situation as disturbed or normal would simplify diagnosis, particularly in large dairy farms. Therefore, this study aimed to evaluate the possible applicability of these serum parameters in the early diagnosis of uterine involution disturbances.

## Materials and Methods

### Ethical approval and Informed consent

The current study was evaluated by the local authority for ethics approval (Regional Board Giessen) and was determined not to require official or institutional ethical approval. Concerning sampling, a notification of the local ethics authority was performed through the animal welfare office of the Justus-Liebig-University Giessen (internal number of correspondence, IRB number: kTV 11-2018).

Blood sampling was performed on the request of the owner due to herd management and herd health diagnostics. Consent to participate in this study was given verbally. The study did not affect the animals in excess of diagnostics and therapy.

### Study period and location

The study was conducted between October 2015 and January 2016 in the Middle-East of Germany (Hessen and Thuringia).

### Animals and study design

In this study, blood samples were collected from 86 dairy cows (aged 2.5-9 years) at six local farms with 54-1254 cows per farm (mean±SD; 406.7±518). At the time of sampling, sample animals were up to 20 days of lactation and were grouped according to the results of the clinical and gynecological examination: Healthy (n=62) or diseased (n=24) groups. Diseased cows were characterized based on the clinical signs presented (vaginal discharge, asymmetry of the uterus, and anestrus) for endometritis, metritis, or involution disturbances due to another cause.

The samples were allowed to clot overnight at 4°C, were subsequently centrifuged for 20 min at approximately 1000× *g*, and stored at −80°C until evaluation. The serum concentrations of SP, VIP, and IL-1β were determined in-house using bovine-specific ELISA kits as per manufacturer’s guideline (USCN Cloud-Clone Corp., Houston TX, USA).

### Statistical analysis

Data analysis involved the testing of peptide blood serum levels of cows at different points in time up to 20 days of lactation and a group comparison between healthy and diseased cows. A regression analysis was conducted to evaluate the time-associated differences of the neuropeptide concentrations. For the group comparison with the covariate time, a one-way analysis of covariance was conducted using the statistical software package BMDP, particularly the program BMDP1V (BMDP Statistical Software; University of California, LA, USA). p<0.05 was considered statistically significant.

Because of biased distribution of IL-1β, IL-1β values were logarithmically transformed in the analysis, and data description is provided using box plots.

As the considerable percentage of values for IL-1β was below the detection limit, time-associated changes and group comparisons for IL-1β were calculated using a one-way analysis of covariance. In addition, the exact version of the non-parametric Wilcoxon–Mann–Whitney test was performed to compare the IL-1β values within the first 10 and 11-20 days of calving (program StatXact^®^, version 9.0.0; Cytel Studio, Cambridge, MA, USA).

## Results

**Figure-1:**
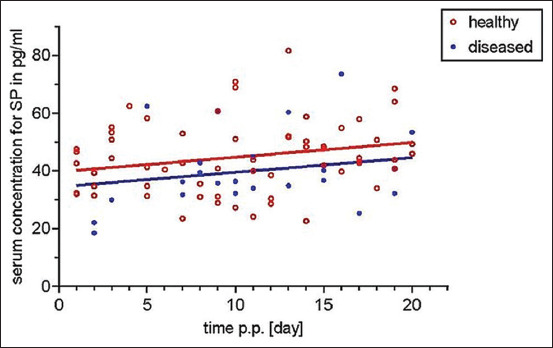
Comparison of serum concentrations of substance P within the first 20 days in milk. Regression lines for healthy (red) and diseased (blue) cows are given. Significant increase of SP concentrations overtime for both groups is shown (p<0.04).

Results showed a statistically significant increase in the serum concentrations of SP within the first 20 days of calving (p<0.036), with no significant slope difference between the groups (p=0.564; Figure-1). No significant difference was found between the adjusted means of the groups (p=0.084).

**Figure-2:**
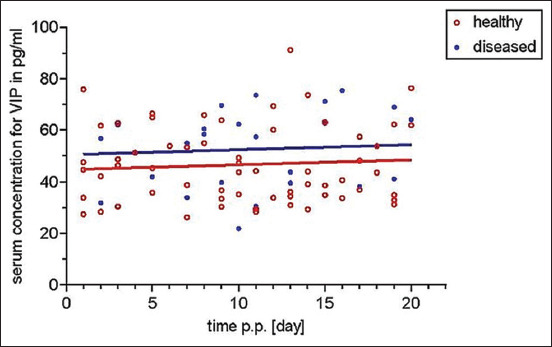
Comparison of serum concentrations of vasoactive intestinal polypeptide within the first 20 days in milk. No significant difference could be detected.

As regards VIP, neither for the timely course (p=0.504) nor for the adjusted group means (p=0.110), significant differences were observed (Figure-2).

**Figure-3:**
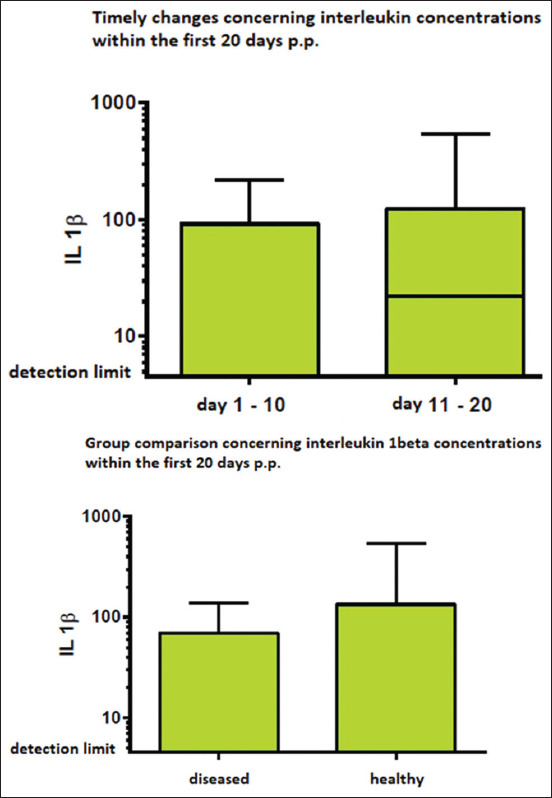
Interleukin (IL)-1β serum concentrations. Left: IL-1β concentrations within the first 10 days in milk in comparison to days 11-20 after calving. Right: Group comparison between healthy and diseased cows within the first 20 days in milk. No statistically significant differences could be detected.

Equivalent findings were obtained for log IL-1β values, with no statistically significant differences found in the timely course (p=0.166) nor within the group comparison (p=0.296; Figure-3). Table-1 shows a detailed overview of the results of the one-way analysis of variance.

**Table-1:** Detailed overview concerning the results of the one-way analysis of covariance.

Parameter	Equality of slopes (p-values)	Common slope		Adjusted group means		
	
		Estimate	p-value	Diseased	Healthy	p-value
SP	0.564	0.51 pg/mL × day	0.036	39.7	44.9	0.084
VIP	0.379	0.19 pg/mL × day	0.504	52.4	46.6	0.110
IL-1β (explorative)	0.205	0.02 pg/mL × day	0.166	1.13	1.31	0.296

SP=Substance P, VIP=Vasoactive intestinal polypeptide IL=Interleukin

The exact Wilcoxon–Mann–Whitney test also yielded no statistically significant differences regarding the timely changes or group comparison for the parameter IL-1β.

## Discussion

Uterine involution disturbances with concurrent fertility problems due to subclinical endometritis are often a reason for therapeutic measures or, even worse, for culling [1]. Therefore, developing reliable and comfortable early diagnostic tools are of utmost urgency. The previous studies have reported lower concentrations of tumor necrosis factor (TNF)-α, IL-1β, IL-6, and IL-8 before calving in cows that developed retained fetal membranes after parturition [8]. In contrast, elevated serum and tissue concentrations of pro-inflammatory cytokines, including TNF-α, IL-1β, and IL-6, were observed in cows with metritis, endometritis, or subclinical endometritis [7]. In cystic ovarian disease, another reason for infertility, cytokine expression was observed to be altered within the follicular structures [10].

This study aimed to clarify whether serum concentrations of IL-1β, SP, and VIP differ time-dependent within the first 20 days of lactation and if these concentrations are correlated to puerperal diseases. The purpose was to clarify if these parameters may be useful for the early diagnosis of uterine disease after calving.

Moreover, based on the results, no statistically significant changes in the concentrations of pro-inflammatory cytokines were shown within the first 20 days of lactation. In contrast to the previous studies, no statistically significant differences were observed between healthy and diseased cows. Nevertheless, when interpreting the results related to IL-1β, the IL-1β concentration in a considerable number of cows, which was under the detection limit (6.4 pg/mL) should be considered. To the best of our knowledge, this finding has not been reported previously. In addition, breed differences might exist concerning the serum concentrations of cytokines. The aforementioned study in cows that were at risk of fetal membrane retention involved Zebu breeds and other Indian crossbreds, wherein retention of the fetal membranes is reported to be more common than in other breeds, such as Holstein-Friesians (HFs) [8]. Nevertheless, the central role of inflammatory processes and placental maturing in the physiological release of fetal membranes have also been shown for HFs [11,12].

However, according to our results, IL-1β is not a suitable diagnostic marker for uterine diseases within the first 20 days of calving.

VIP also seems to be of no significance either for uterine involution or for uterine health. Our results revealed no significant differences, according to time after calving or healthy or diseased condition. A study on VIP within the reproductive tract only refers to tissue distribution, not serum concentrations [13].

In contrast, as regards SP, statistical analyses revealed significant time-dependent differences within the first 3 puerperal weeks. SP and VIP typically are present within the vaginal and uterine tissues of cows [13]; particularly, the role of SP is postulated in cervical softening and birth [14]. To the best of our knowledge, this study is the first to describe a time-related change in SP serum concentrations after calving. Increasing concentrations of this neuropeptide might be associated with uterine involution. Although the group comparison between healthy and diseased animals showed a barely significant difference, the difference may be significant if the number of samples studied could be increased. Future studies should, therefore, focus on the establishment of normative values related to breed, age, lactation status, and pregnancy, because previously published literature only refers to SP concentrations in calves [15]. Another point of criticism is that the concentrations of IL or neuropeptides were not examined continuously in the same animal. Results might have been influenced by the time of sampling, the sampling collection itself, or the time from collection to examination. However, the study design did not intend to examine changes over time or the influence of associated factors but the usability of single-shot sampling in the diagnosis of early puerperal diseases under field conditions.

## Conclusion

Neither the pro-inflammatory cytokine IL-1β nor the neuropeptide VIP is proposed as a suitable single test indicator of uterine involution disturbances or uterine diseases within the first 3 weeks of calving. Concerning the neuropeptide SP, time-associated differences were observed in serum concentrations after calving, and a correlation to diseases might not be precluded. Further research is needed regarding the establishment of normative values concerning this parameter. Timely changes and age- or parity-dependent differences should explicitly be considered. Due to its involvement as a transmitter of pain and inflammation through sensible neurons, SP concentrations also might serve as an indicator for the necessity of anti-inflammatory therapy.

## Authors’ Contributions

AW, IJ, and MS: Conceptualization. AA: Performed sampling and generated data. KF and JR: Performed the statistical analysis. MS: Drafted the manuscript. AW and MS: Reviewed and edited the manuscript. All authors read and approved the final manuscript.

## Data Availability

The data analyzed during the present study are available from the corresponding author on reasonable request.
